# AGO2 and SETDB1 cooperate in promoter-targeted transcriptional silencing of the androgen receptor gene

**DOI:** 10.1093/nar/gku788

**Published:** 2014-09-02

**Authors:** Sunwha Cho, Jung Sun Park, Yong-Kook Kang

**Affiliations:** 1 Development and Differentiation Research Center, KRIBB, 111 Gwahangno, Yuseong-gu, Daejeon 305-806, South Korea; 2 Department of Functional Genomics, University of Science and Technology (UST), 113 Gwahangno, Yuseong-gu, 305-333 Daejeon, South Korea

## Abstract

In mammals, RNA interference is primarily a post-transcriptional mechanism. Evidence has accumulated for additional role in transcriptional gene silencing (TGS) but the question for a good paradigm for small interfering antigene RNA (agRNA)-induced chromatin modification remains unanswered. Here, we show that SETDB1, a histone H3-lysine 9 (H3K9)-specific methyltransferase, cooperates with Argonaute-2 (AGO2) and plays an essential role in agRNA-induced TGS. The androgen receptor (*AR*) gene was transcriptionally silenced by agRNA targeted to its promoter, and we show that this repression was mitigated by knockdown of *SETDB1* or *AGO2*. Chromatin immunoprecipitation demonstrated that agRNA-driven AGO2 was first targeted to the *AR* promoter, followed by SETDB1. SIN3A and HDAC1/2, the components of the SIN3-HDAC complex, immunoprecipitated with SETDB1, and localized at the agRNA-targeted promoter. Agreeing with the presence of SETDB1, trimethyl-H3K9 was enriched in the *AR* promoter. Both EZH2 and trimethyl-H3K27 were also present in the targeted locus; accordingly, EZH2 immunoprecipitated with SETDB1. DNA methylation level was not significantly changed, suggesting the absence of *de novo* methylating activity in agRNA-induced *AR* promoter. Our results demonstrate that SETDB1, together with AGO2, plays an essential role in TGS through recruiting chromatin remodeler and/or other modifiers, consequently creating a repressive chromatin milieu at the targeted promoter.

## INTRODUCTION

RNA interference (RNAi), first established in 1998 by Waterhouse *et al.* (1) and Fire *et al*. (2), denotes small RNA-mediated silencing. It functions in the cellular control of gene expression and protects the genome against mobile repetitive DNA sequences (3–5). Small silencing RNAs are characterized by their short length (20–30 nucleotides) and their association with Argonaute (AGO) proteins. The small RNA-AGO complex establishes the RNA-induced silencing complex (RISC) (6) and act as specificity factors to target homologous sequences for repression (7). RNAi frequently acts at the post-transcriptional level, reducing gene expression by directing transcript cleavage or translational inhibition. In addition to these well-known roles in post-transcriptional gene silencing (PTGS), RNAi can also trigger chromatin modifications (DNA methylation and/or histone modifications) that lead to heterochromatin formation and transcriptional gene silencing (TGS) in the nucleus.

The TGS mechanism is best described for *Schizosaccharomyces pombe*, in which Ago proteins bind Dicer-generated siRNAs and the accessory proteins Chp1 and Tas3, forming the RNA-induced transcriptional silencing (RITS) complex and resulting in direct or indirect histone H3 lysine 9 methylation (H3K9me) at pericentromeric sequences (reviewed in (8,9)). Clr4 (cryptic loci regulator 4), the sole H3K9 methyltransferase in *S. pombe*, mediates H3K9me synthesis, creating binding sites for the chromodomain proteins Swi6, Chp1 and Chp2, as well as Clr4 itself (10–12). Clr4 binds Dos1, Dos2, Rik1 and Cul4 to form the CLRC (cryptic loci regulator complex) (13–16), and the CLRC associates with the RITS complex on nascent transcripts via Stc1, demonstrating a mechanism of coupling RNAi to chromatin modifications in *S. pombe*.

In mammalian cells, the mechanism of TGS is not as well established as PTGS. There is increasing evidence that TGS can also suppress gene transcription, but it is not clear what primary function this serves. Early examples of TGS came from exogenous double-stranded RNAs (dsRNA). Since the initial usage of inverted-repeat transgenes to produce siRNAs homologous to a target promoter (17) and the discovery of transgene- and viral RNA-guided DNA methylation of homologous sequences in plants (18), many studies across phyla (19–23) have reported that small dsRNA, or antigene RNA (agRNA), complementary to the promoter can elicit TGS and inhibit gene expression, establishing agRNAs as central players in RNA silencing pathways (reviewed in (8–9,24–25)). More recently, it was observed that siRNAs directed to intragenic sequences can regulate alternative splicing (26,27). The TGS process, targeting either promoters or exonic/intronic sequences, is accompanied by recruitment of chromatin-modifying proteins in human cells, which entails H3K9 dimethylation (H3K9me2), H3K27 trimethylation (H3K27me3), histone deacetylation and/or DNA methylation to transform target loci to repressive heterochromatin (20,28–29). Additionally, it was recently shown that AGO2, RB1 and *let-7* miRNA physically interact to establish senescence-associated TGS at RB1/E2F target genes via repressive chromatin modifications, involving H3K9me2 and H3K27me3, at the promoters (30). The above observations demonstrate that AGO2-mediated TGS is involved in diverse cellular processes in mammals and that in these processes, silent-state chromatin modifications are introduced to target regions, as in *S. pombe*. However, in mammals, the components of the RITS complex in TGS and which chromatin modifying activity collaborates on local silencing with the RITS complex, are yet to be defined. In *S. pombe*, Clr4 is the only H3K9-specific methyltransferase but in humans, Clr4-clan enzymes with H3K9 specificity exist redundantly (SUV39H1 (31), G9A (32), GLP (33) and SETDB1 (34)), and it is unclear which enzyme(s) function in small agRNA-induced TGS.

SETDB1 was identified through its interaction with the ETS transcription factor ERG (35), and various transcriptional regulators have been found to be associated with SETDB1. SETDB1 interacts with transcriptional repressors, such as KAP-1 (36), HDAC1/2 and mSin3A/B (37), Pml (38,39) and Sp3 (40), and contributes to heterochromatin protein 1 (HP1)-mediated formation of facultative heterochromatin (41–43). SETDB1 is involved in establishing heterochromatin structure during germ cell development in *Drosophila* (44), in the maintenance of heterochromatin structure and during DNA replication in mammals via association with MBD1 and CAF-1 (45). Therefore, the wide-ranging involvement of SETDB1 in the formation of heterochromatin in mammalian cells hints that SETDB1 may have an important role in chromatin modification in agRNA-triggered TGS.

In this study, we investigated the participation of SETDB1 in *de novo* formation of heterochromatin in agRNA-directed TGS. Several observations implicate SETDB1 in this process; SETDB1 synthesizes trimethylated H3K9 (H3K9me3) and recruits HP1, instrumental in establishing and maintaining heterochromatin (46,47). The role of SETDB1 in establishing heterochromatin in various biological systems has been previously demonstrated (43–45,48–50), and heterochromatin can be formed via an RNAi-mediated mechanism (25). Furthermore, as we recently reported, SETDB1 may reside partially in the cytoplasm in certain cells (51), where the RNAi mechanism is initiated. We used a well-established expression analysis system for the androgen receptor (*AR*) gene in T47D cells to test the hypothesis that SETDB1 collaborates with AGO2 to transcriptionally repress *AR* gene expression.

## MATERIALS AND METHODS

### Antibodies

The list of antibodies we used was as follows: anti-SETDB1 (Upstate 07-378 for NT and Abcam ab12317 for CT), -DICER1 (sc-30226, Santa Cruz), -AGO2 (2897, Cell signaling), -AR (3202, Cell signaling), -PR (3176, Cell signaling), -β-actin (sc-47778, Santa Cruz), -KAP1 (ab10483, Abcam), -EZH2 (3147, Cell signaling), -SIN3A (ab129087, Abcam), -HDAC1 (sc-7872, Santa Cruz), -HDAC2 (sc-7899, Santa Cruz), -MTA2 (ab8106, Abcam), -DNMT3A (D23G1, Cell signaling) and -DNMT3B (ab13604, Abcam).

### Cell culture, transfection and reverse transcription-polymerase chain reaction (RT-PCR)

T47D cells were maintained in RPMI-1640 media (Gibco) supplemented with 10% fetal bovine serum (FBS), 0.5% non-essential amino acids, 0.4 units/ml bovine insulin, 100 units/ml penicillin and 0.1 mg/ml streptomycin. Culture was kept in a humidified atmosphere with 5% CO_2_ at 37°C. For knockdown experiments, we used the same duplex agRNAs, AR50 and PR26, and control RNA duplex with an arbitrary sequence, MM1, that were used in a previous study (52) (see also Supplementary Table S1). AGO2 and SETDB1 knockdown constructs were purchased from Dharmacon. For transfection, cells were plated in 6-well plates 2 days before transfection and transfected with 25 nM duplex RNA per well using RNAi-MAX (Invitrogen) and cells were harvested 5 days after transfection.

For RT-PCR, total RNAs were obtained using RNeasy mini kit (Qiagen). Two micrograms of total RNAs were used for cDNA synthesis. cDNA was synthesized using oligo-dT primer and moloney murine leukemia virus (MMLV) reverse transcriptase (SuperScript II, Invitrogen) according to the manufacture's instruction. Primers used for detection of *AR*,*PR* and *GAPDH* transcripts are listed in Supplementary Table S1. Quantitative real-time PCR was performed on the ABI-7500 Real-Time PCR System (Applied Biosystems) using TOPreal qPCR 2× PreMIX (Enzynomics). To detect AR non-coding RNA (ncRNA), 1 μg of total RNAs from T47D cells were used in cDNA synthesis using each of promoter-specific primers (F1-F4 and R1-R4, see Supplementary Table S1). For normalization of the level of *AR* ncRNA, GAPDH cDNA was concurrently synthesized using a gene-specific primer (5′-AGTGATGGCATGGACTGTGG-3′) annealing to *GAPDH* mRNA. With the synthesized cDNAs as templates, PCR was performed using sets of primers indicated (Supplementary Table S1).

### Immunoprecipitation (IP) and chromatin immunoprecipitation (ChIP)

Cells were treated with a lysis buffer (0.5% NP-40, 50mM Tri-Cl, 10% glycerol, 0.1 M ethylenediaminetetraacetic acid (EDTA), 15 mM NaCl) for 1 h to collect whole cell extracts. Note that 10 μg of anti-SETDB1 (NT) antibody was incubated with 100 μg of whole cell extracts for overnight at 4°C on a rotator. Note that 50 μl of protein A or protein A/G beads were then added and incubated another 4 h at 4°C. Beads were washed and boiled in 50 μl of 2× sodium dodecyl sulphate (SDS) sample buffer (100 mM Tris-Cl, pH 6.8, 2% SDS, 20% glycerol, 0.02% bromophenol blue, 4% β-mercaptoethanol) for 5 min. Note that 20 μl of the supernatant were resolved on a SDS-polyacrylamide gel electrophoresis gel for western blot analysis. For ChIP, cells were first fixed with 1% formaldehyde at room temperature for 10 min before being stopped by glycine (to 0.125 M of final concentration). ChIP was then performed using 10 μg of indicated antibodies (39). ChIP products were used as templates in quantitative real-time PCR to measure target enrichment. H3K9me, H3K27me3, EZH2, Pol II and AGO2 ChIP products were used in semi-quantitative PCR, and the band density of the PCR products was determined with densitometry (TINA20). Sets of primers used in ChIP PCR are listed in Supplementary Table S1. All IP and ChIP experiments were duplicated or triplicated to verify results.

### RNA IP (RIP)

agRNA-transfected cells were cross-linked with 1% formaldehyde at room temperature for 10 min, and the reaction was stopped by glycine (to 0.125 M of final concentration). The samples were washed with ice-cold phosphate buffered saline and lysed in RNA immunoprecipitation (RIP) buffer (25 mM Tris-HCl at pH 7.4, 150 mM KCl, 5 mM EDTA, 0.5 mM DTT and 0.5% NP-40) containing protease inhibitors (GenDEPOT) and RNaseOUT (Invitrogen) for 1 h on ice. The suspension was sonicated and centrifuged to remove insoluble materials. The supernatants were incubated overnight with anti-SETDB1 antibody at 4°C. Note that 50 μl of protein A or protein A/G beads were incubated for additional 4 h at 4°C, and the beads were washed with RIP buffer. RNAs were eluted for 20 min at 37°C in 150 μl of RIP Elution buffer (10 mM EDTA, 100 mM Tris-HCl at pH 8.0, 1% SDS) containing 40 units/ml RNaseOUT. Eluted RNA and 10% input fraction were incubated with final concentration of 200 mM NaCl for 1 h at 65°C to reverse the cross-linking. RNA was extracted using RNeasy mini columns (Qiagen) according to the manufacturer's instructions and then incubated either with DNase I (Takara) to avoid DNA contamination or with DNase I plus RNase A to obtain a negative control sample for 10 min at 37°C. cDNA was synthesized using promoter-specific primer and SuperScript II (Invitrogen) according to the manufacture's instruction. With the synthesized cDNA as templates, quantitative RT-PCT (qRT-PCR) was performed on the ABI-7500 Real-Time PCR System (Applied Biosystems) using TOPreal qPCR 2× PreMIX (Enzynomics).

### Fractionation of nuclear and cytoplasmic proteins

For separation of nuclear proteins from cytoplasmic proteins, cell pellets were suspended in a hypotonic solution (10 mM HEPES-KOH, pH 7.9, 10 mM KCL, 0.1 mM EDTA, 1 mM DTT, 1× protease cocktail, 1 mM phenylmethanesulfonyl fluoride (PMSF)) and incubated at 4ºC for 10 min before centrifugation at 1500 × *g* for 5 min at 4ºC. Supernatant was collected as cytoplasmic fraction. The remaining pellets were carefully suspended in 1 ml of hypotonic solution containing 30% sucrose, centrifuged at 13 600 × *g* for 10 min at 4ºC and incubated in 150 μl of a high-salt solution (20 mM HEPES-KOH, pH 7.9, 400 mM NaCl, 1 mM EDTA, 10% glycerol, 1 mM DTT, 1× protease cocktail, 1 mM PMSF) for 1 h at 4ºC. Pellets were centrifuged at 13 600 × *g* for 10 min at 4ºC and the proteins were harvested as nuclear fraction.

### Bisulfite sequencing

Genomic DNA was isolated and treated with bisulfite (53) using EpiTect kit (Qiagen) according to the manufacturer's protocol. The bisulfite-treated DNA was amplified with 21 cycles of 94°C for 30 s, 50°C for 40 s and 72°C for 30 s in the first PCR and then another 31 cycles of 94°C for 30 s, 55°C for 40 s and 72°C for 30 s in the nested PCR. Primers used are listed in Supplementary Table S1. The PCR products were purified and ligated into pGEM-T easy vector (Promega). PCR was performed three times separately before pooling and sequencing.

## RESULTS

### SETDB1 is necessary for AGO2-mediated TGS

**Figure 1.**
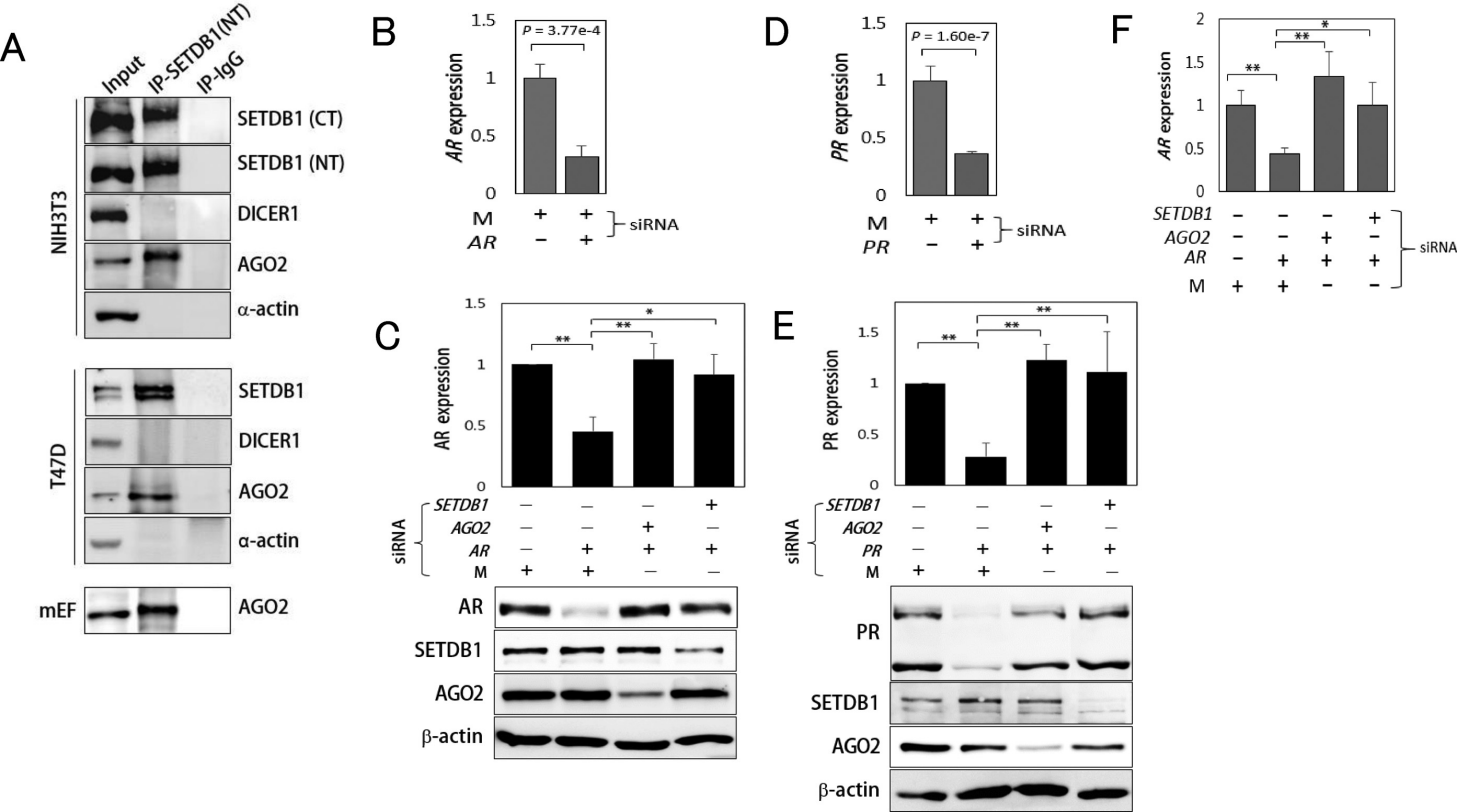
SETDB1 and AGO2 cooperate in agRNA-induced transcriptional silencing of AR and PR gene expression. (**A**) IP using α-SETDB1. Immunoprecipitated products were probed for the indicated proteins. Cells used for IP are designated. mEF, mouse embryonic fibroblasts; T47D, human ductal breast epithelial tumor cell. Two different anti-SETDB1 antibodies were used: NT and CT antibodies recognizing amino- and carboxyl-terminal regions of SETDB1, respectively (51). (**B–F**) agRNA-mediated repression of *AR* (B, C and F) and *PR* (D and E) gene expression at the mRNA (B, D and F) and protein level (C and E) in T47D cells. All experiments were performed at least twice. Error bars and standard deviations are shown. M, duplex RNA with an arbitrary sequence, was used as a control (52). In C, E and F, one-way ANOVA and the Bonferroni *post hoc* test were used for statistical analysis. Single (*P* < 0.05) and double asterisks (*P* < 0.01) denote a significant difference between samples.

As the main component in the RNAi mechanism, AGO2 processes dsRNAs from DICER1 into siRNAs (25,46,54) and thus is the catalytic engine of RISC (55). First, we used IP to determine whether SETDB1 associates with AGO2. AGO2, but not DICER1, was indeed precipitated using an anti-SETDB1 antibody in extracts from mouse NIH3T3 and embryonic fibroblasts and human T47D cells (Figure 1A).

We tested whether SETDB1 participates in AGO2-mediated silencing of target gene expression. AGO2 was previously shown to be recruited to the target site in the presence of agRNAs complementary to the promoter sequences of the *AR* and progesterone receptor (*PR*) genes to initiate TGS (52). Using the same experiments and agRNAs used in the previous study, we confirmed that the expression of *AR* and *PR* genes were reduced in T47D cells both at the transcriptional (Figure 1B and D) and translational level (Figure 1C and E). This effect vanished when joint siRNAs, such as [*AR+AGO2*] or [*PR+AGO2*], were introduced (Figure 1C and E), in agreement with a previous study (52). Interestingly, [*AR+SETDB1*] or [*PR+SETDB1*] knockdown also eliminated silencing from *AR* and *PR* only knockdown. *AGO2* knockdown did not affect SETDB1 protein levels in T47D cells, and vice versa. qRT-PCR showed that *AR* expression was decreased only with *AR* agRNA alone (Figure 1F). Therefore, the results suggest that in the absence of *AGO2* or *SETDB1*, agRNA-induced TGS fails to be induced at *AR* and *PR* promoters.

### agRNA-targeted *AR* promoter is modified by SETDB1-catalyzed H3K9me3 and EZH2-catalyzed H3K27me3

**Figure 2.**
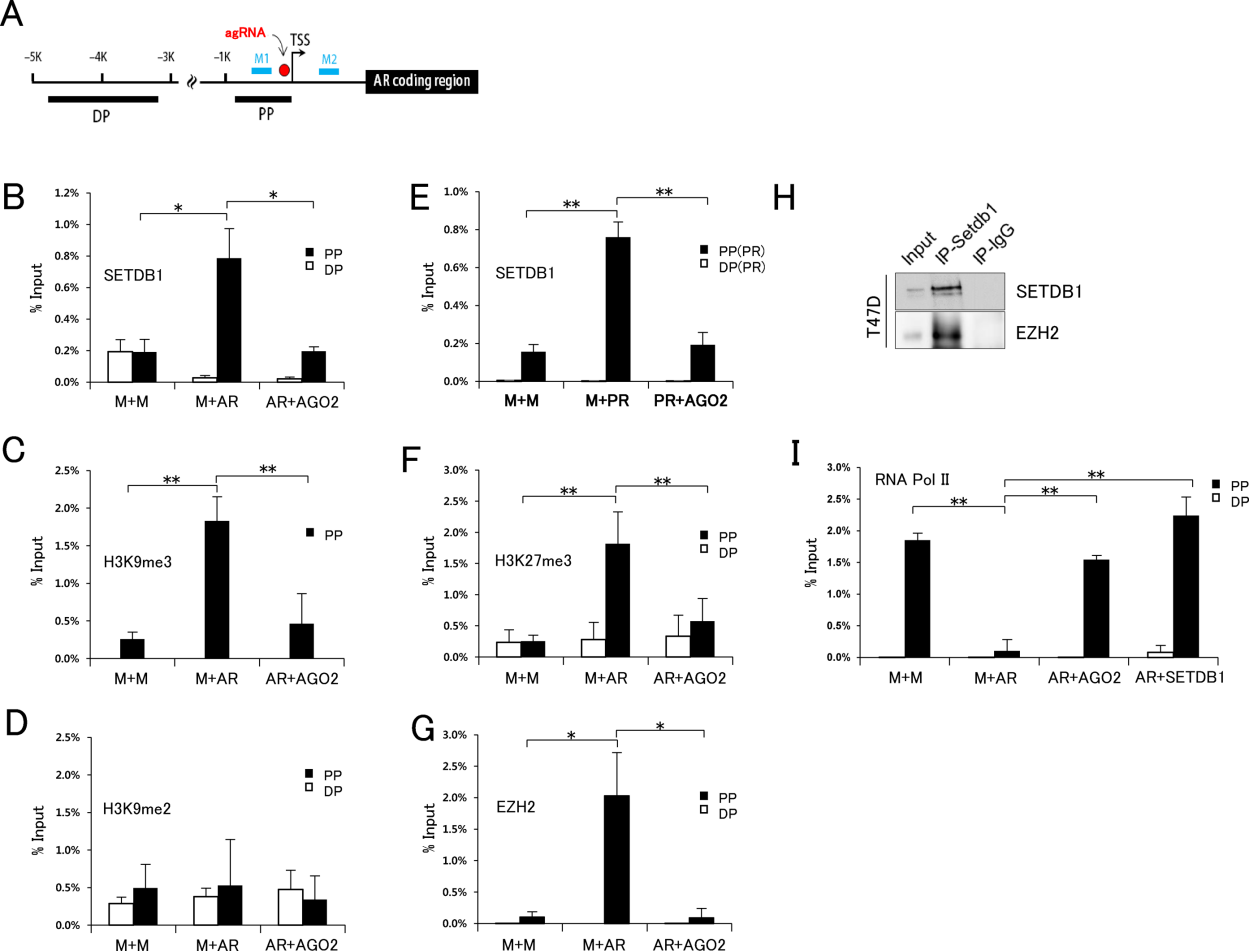
Protein recruitment and histone modifications at the AR promoter upon agRNA-induced transcriptional silencing of the AR gene. (**A**) *AR* promoter region used in ChIP [proximal promoter (PP) and distal promoter (DP)] and DNA methylation analysis (M1 and M2, see Figure 4). TSS, transcriptional start site. Distance from the TSS (TSS; +1) is designated in kb. The agRNA target site is indicated with a circle. (**B–H**) ChIP using indicated antibodies in T47D cells transfected with indicated siRNAs. In E, progesterone (*PR*) promoter region was analyzed in ChIP-PCR. Panel H shows IP of EZH2 in T47D cells using α-SETDB1 antibody. One-way ANOVA and the Bonferroni *post hoc* test were used for statistical analysis. Single (*P* < 0.05) and double asterisks (*P* < 0.01) denote a significant difference between samples.

We next looked for SETDB1 at the targeted *AR* promoter through ChIP using an anti-SETDB1 antibody. By PCR, SETDB1 was shown enriched at the *AR* proximal promoter but not at the distal promoter (Figure 2A and B). However, in the [*AR+AGO2*] combined knockdown, SETDB1 was not enriched at the *AR* promoter. Correlating with the presence of SETDB1, H3K9me3, the enzymatic product of SETDB1 and a typical heterochromatin marker (10,56), emerged *de novo* in the promoter region (Figure 2C), and this increased H3K9me3 was not seen upon [*AR+AGO2*] combined knockdown. However, H3K9me2 was not enriched in the agRNA-targeted promoter region (Figure 2D). We further examined whether SETDB1 was also involved in TGS of the *PR* gene promoter. SETDB1 ChIP results showed that SETDB1 localized to the targeted *PR* promoter (Figure 2E). However, it was not detected under the [*PR+AGO2*] combined knockdown condition. The similarity in the behavior of SETDB1 toward the targeted *AR* and *PR* promoters suggests that SETDB1 plays a general role in agRNA-induced TGS.

In previous studies, promoter-targeted TGS was associated with H3K27me3 (22,57). We confirmed that H3K27me3 was enriched in the *AR* promoter in *AR* knockdown cells, but not in [*AR+AGO2*] knockdown cells (Figure 2F). Supportively EZH2, which is responsible for synthesis of H3K27me3 and forms the polycomb repressor complex 2 (PRC2), was similarly enriched at the targeted promoter region (Figure 2G). EZH2 associates with SETDB1 based on IP experiments (Figure 2H), suggesting a collaborative mechanism between H3K9me3 and H3K27me3 marks in agRNA-induced TGS.

H3K9me3 and H3K27me3 enrichment is correlated with gene silencing (58). However, RNA polymerase II (Pol II) was present at a substantial fraction of H3K27me3-enriched promoters (59), from which only low transcript levels were detected, though (60), indicating that Pol II may be paused at PcG-targeted genes (61). We examined whether Pol II occupied the H3K27me3-abundant *AR* promoter. The *AR* promoter was vacant in *AR* knockdown cells by ChIP analysis, whereas Pol II remained bound in [*AR+AGO2*] and [*AR+SETDB1*] combined knockdown cells (Figure 2I). So, we assume that Pol II has difficulty in positioning at the promoter when the promoter is simultaneously modified with H3K9me3 and H3K27me3.

### AGO2 recruits SETDB1 and the SIN3-HDAC complex for agRNA-mediated transcriptional silencing of the *AR* promoter

**Figure 3.**
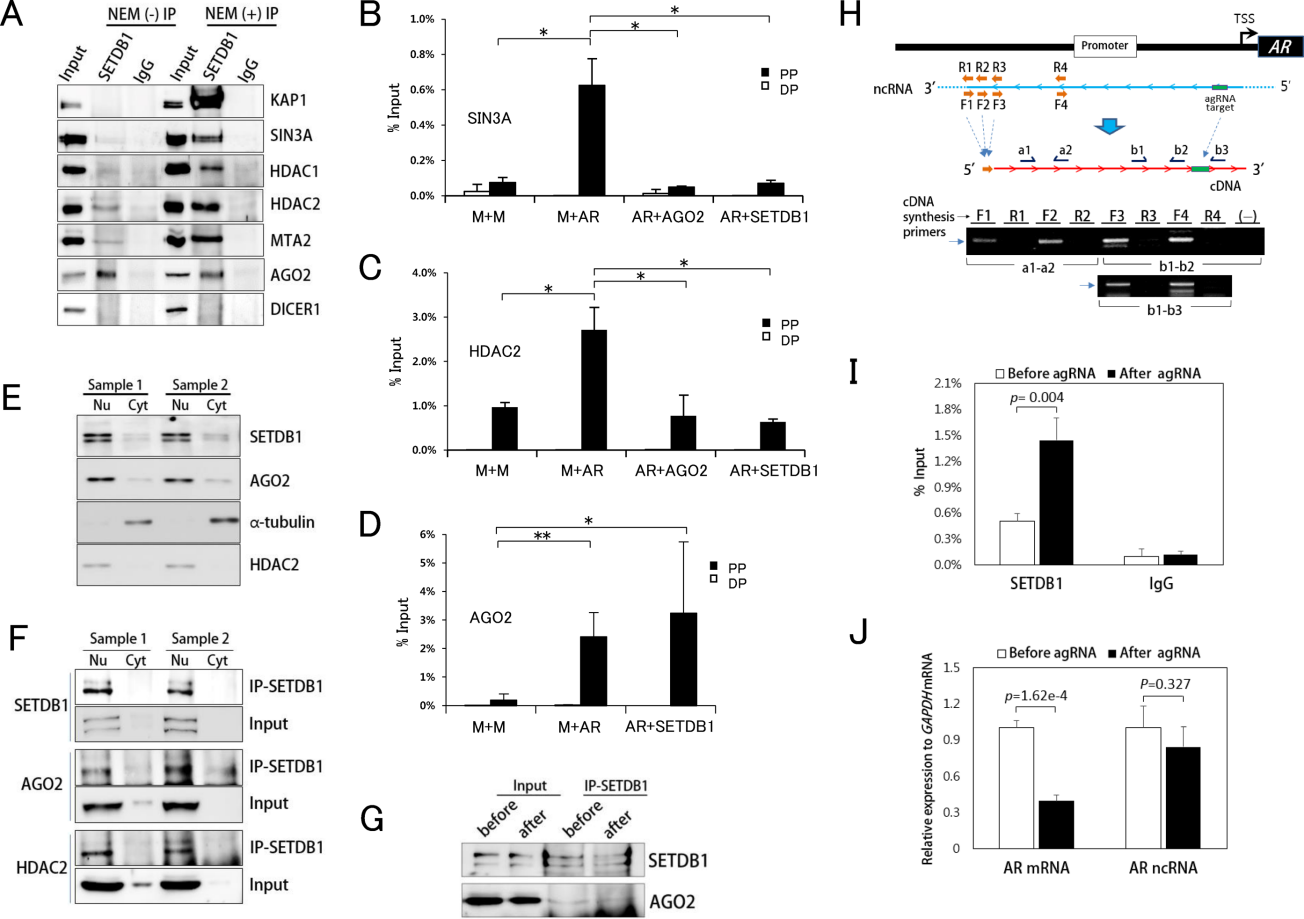
AGO2-SETDB1 complex associates with AR gene non-coding RNA at targeted promoter and recruits the SIN3-HDAC chromatin remodeling complex. (**A**) IP using an α-SETDB1 antibody. T47D cell lysates were obtained in the presence (+) or absence (-) of NEM that is a SENP inhibitor known to stabilize SUMO conjugation. DICER1, negative control (see Figure 1A). (**B–D**) ChIP in T47D cells after dsRNA transfection using indicated antibodies. PP and DP, proximal and distal promoter, respectively. One-way ANOVA and the Bonferroni *post hoc* test were used for statistical analysis. Single (*P* < 0.05) and double asterisks (*P* < 0.01) denote a significant difference between samples. (**E** and **F**) Protein fractionation (E) and IP with fractioned proteins using α-SETDB1 antibody (F) in T47D cells. In E, α–tubulin and HDAC2 serve as a nuclear and a cytoplasmic marker, respectively. Nu, nuclear fraction; Cyt, cytoplasmic fraction. (**G**) IP with normal cell lysates (before) or lysates from agRNA-transfected cells (after) using α-SETDB1 antibody. (**H**) Detection of *AR* non-coding RNA expression. cDNAs were synthesized using either a specific sense primer (F1, F2, F3 or F4) that is annealed to antisense non-coding RNA (linear blue line) or its complementary primer (R1, R2, R3 or R4, respectively) as a negative control. PCR products were detected only from the cDNAs synthesized by the sense primers. Primer sets (a1-a2, b1-b2 and b1-b3) for PCR are indicated. PCR with the b1-b3 primers, which can amplify the region encompassing the *AR* agRNA target, employed cDNA templates synthesized using F3, R3, F4 or R4 primer. TSS, transcription start site; gDNA, genomic DNA; (-), no template. (**I**) RIP using α-SETDB1 antibody. SETDB1 IP specifically retrieves *AR* ncRNA. Mock IP using immunoglobulin G is also shown. (**J**) Comparison of the level of *AR* non-coding RNA before and after the *AR* agRNA treatment. With precipitated RNAs (I) or total RNAs (J), cDNA was synthesized using F3 primer and then quantitative real-time PCR was performed using b1-b3 primer set. For normalization of the level of *AR* ncRNA in J, *GAPDH* cDNA was concurrently synthesized using a gene-specific primer annealing to *GAPDH* mRNA. As a reference, the level of *AR* mRNA is also compared in the same cell preparation. Statistics in I and J, *t*-test.

We next investigated which chromatin remodeling complex is involved in AGO2-SETDB1-mediated *AR* gene silencing. A number of proteins, including KAP1, SIN3A, HDAC1, HDAC2 and MTA2, are associated with SETDB1 by IP (Figure 3A). For SIN3A, HDAC1, HDAC2, KAP1 and MTA2, the efficiency of IP was greatly improved with the addition of *N*-ethylmaleimide (NEM) during cell lysis. Since NEM prevents cleavage of SUMO from proteins and thus stabilizes SUMO conjugates (62), these proteins may interact with SETDB1 via SUMO. In agreement with the above data, interaction of SETDB1 and KAP1 via SUMO has been previously reported (63). This is not the case with AGO2; to put it the other way, SETDB1 may interact with AGO2 in a different fashion, for example, through a direct binding without SUMO mediation or an indirect association via unknown intermediary.

We then tested whether these proteins could bind the *AR* agRNA-targeted promoter via ChIP analysis. SIN3A and HDAC2, key members of the SIN3-HDAC corepressor complex (64), were enriched at the targeted promoter (Figure 3B and C, respectively). SIN3A and HDAC2 were not associated with the *AR* promoter under [*AR*+*AGO2*] or [*AR*+*SETDB1*] knockdown conditions, suggesting that binding to the targeted promoter is dependent on the presence of AGO2 and SETDB1. Our results indicate that the SIN3A-HDAC corepressor aids AGO2 and SETDB1 in modifying the chromatin surrounding the targeted promoter to a transcriptionally inactive state. In mice, ESET (mouse version of SETDB1) was reported to interact with mSin3A/B and Hdac1/2 in cultured cells (37). A number of studies have shown that KAP1 partners with SETDB1 for gene repression, and we also observed this interaction in T47D cells (Figure 3A). However, ChIP experiments did not show association of KAP1 (or MTA2) at the *AR* promoter (data not shown), suggesting that SETDB1-KAP1 and SETDB1-MTA2 interactions are not localized at the targeted *AR* promoter in T47D cells.

AGO2 is present in both the cytoplasm and the nucleus (65). SETDB1 functions mainly in the nucleus but can also localize in the cytoplasm, depending on the type of cells (51,66). As shown in Figure 3D, *AR* agRNA-bound AGO2 was localized at the promoter even in the absence of SETDB1 with AGO2 alone remaining and the *AR* promoter still active (Figure 1C), suggesting that AGO2 arrives first and then recruits nuclear SETDB1. Meanwhile, we observed that SETDB1 and AGO2 localized mainly in the nucleus in T47D cells (Figure 3E). IP experiments with nuclear and cytoplasmic fractions showed that SETDB1 interacted with AGO2 and HDAC2 in the nucleus, verifying that TGS is a nuclear process (Figure 3F). In addition, when SETDB1 IP was performed with normal cell lysates or lysates from agRNA-transfected cells, no difference in the amount of immunoprecipitated AGO2 was detected (Figure 3G), suggesting that AGO2-SETDB1 interaction is not agRNA-specific.

### Detection of *AR* non-coding RNA in association with SETDB1-AGO2 protein complex

We then searched an antisense non-coding RNA (ncRNA) expressed within the *AR* genomic regions that harbors a region complementary to the *AR* agRNA. With RT-PCR, this ncRNA was detected when cDNA was synthesized using a set of primers designed to specifically anneal to the antisense ncRNA (Figure 3H). Sequencing confirmed that the resulting RT-PCR products contained the *AR* promoter sequence. When we searched this ncRNA in several long non-coding RNA databases including GENCODE v19 (http://www.gencodegenes.org) and lncRNA Database (http://www.lncrnadb.org), we could not find it in them, indicating that this is the first to report the *AR* ncRNA. Experiments such as 5′-rapid amplification of cDNA end (RACE) and 3′-RACE PCR to identify the start and end sites of the ncRNA help us better understand about this ncRNA.

We further examined whether the *AR* ncRNA was immunoprecipitated with SETDB1-AGO2 complex. RIP using α-SETDB1 antibody showed that the *AR* ncRNA was in association with SETDB1-AGO2 protein complex in cells transfected with the agRNA (Figure 3I). We then compared the levels of ncRNA before and after agRNA treatment. The *AR* mRNA level was significantly reduced after agRNA treatment, but the expression of the ncRNA was not greatly changed (*P* = 0.327; Figure 3J). This result precludes the possibility that transcription of the *AR* ncRNA is shut down by agRNA-directed TGS of the *AR* promoter or that the ncRNA is targeted for agRNA-directed cleavage by AGO2.

### DNA methylation does not participate in TGS of the *AR* promoter

**Figure 4.**
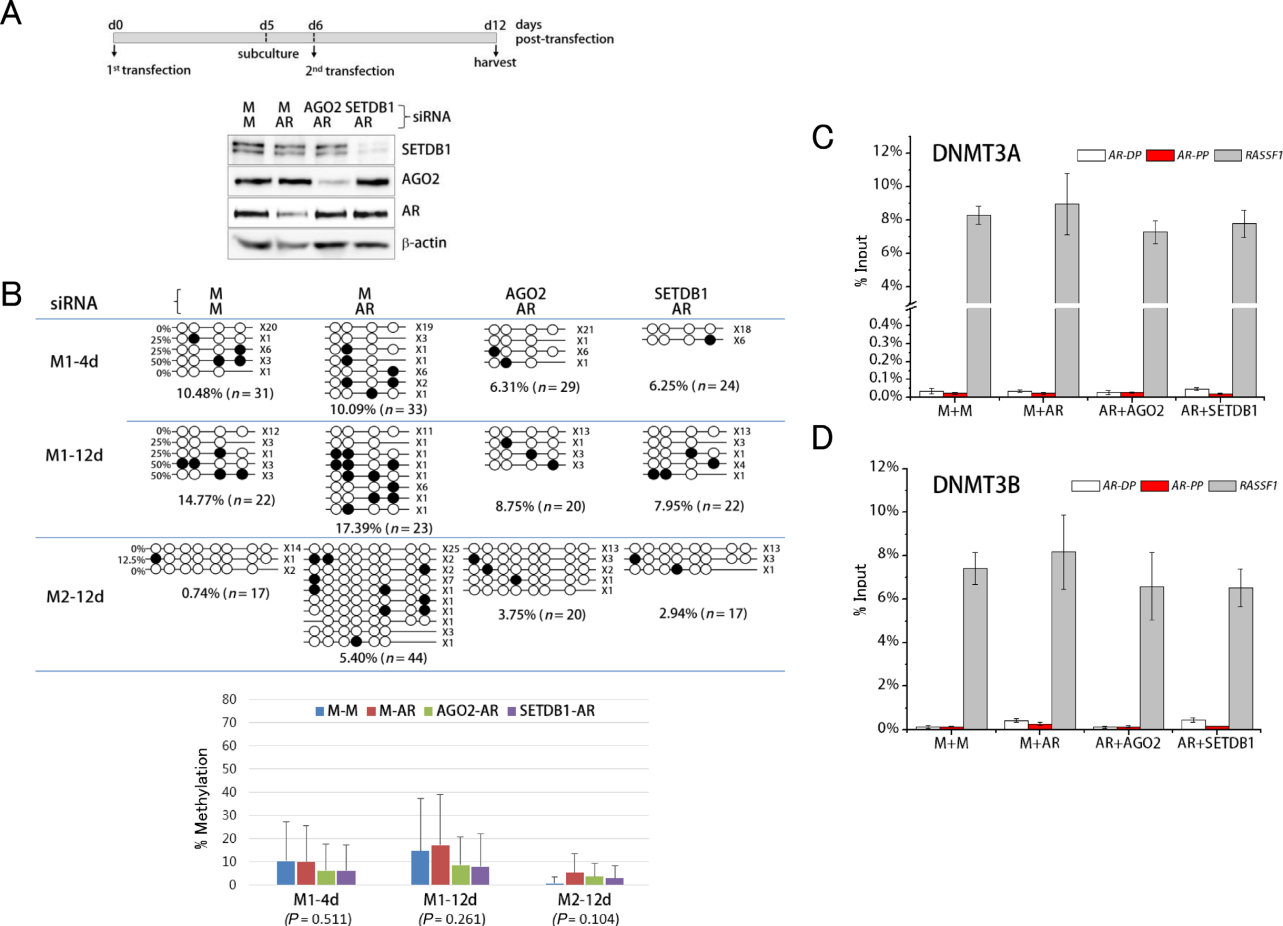
DNA methylation in the agRNA-targeted AR gene promoter. (**A**) Transfection strategy for prolonged maintenance (12 days) of transcriptional silencing at the *AR* gene promoter. (**B**) Bisulfite mutagenesis. Four (4d) and 12 days (12d) after siRNA transfection, T47D genomic DNA was extracted and treated with sodium bisulfite (53). Filled circles denote methylated cytosines and open circles unmethylated cytosines in CpG dinucleotides. Methylation states are shown on PCR strings with the number of clones on the right. Methylation levels (mean ± standard deviation) of the four dsRNA-transfected samples in M1–4d, M1–12d and M2–12d groups are graphically presented below with *P*-value in parenthesis (one-way ANOVA). (**C** and **D**) ChIP in T47D cells after dsRNA transfection using α-DNMT3A (C) and α-DNMT3B (D). *AR*-PP and *AR*-DP, proximal and distal promoter of *AR* gene, respectively. RASSF1 promoter region was included as a positive control in ChIP experiment.

We next examined the methylation state of CpG dinucleotides at the silenced *AR* promoter using bisulfite mutagenesis. To observe methylation changes over a wider window of time, we attempted a repeated transfection based on the schedule shown in Figure 4A, and confirmed that the knockdowns persisted during the period. The CpG methylation profile in Figure 4B showed that the M1 and M2 regions (see Figure 3A) were overall undermethylated, regardless of the presence of *AR* agRNA. This low methylation level was not greatly changed when we lengthened the duration of knockdown up to 12 days. When one-way ANOVA was applied to all of the data sets (M1-4d, M1-12d and M2-12d), no statistical difference in methylation level at *P* < 0.05 was found among the four dsRNA-transfected cell groups (Figure 4B).

We further examined whether DNA methyltransferase activity was involved in agRNA-induced TGS. DNA methyltransferase 3A (DNMT3A) was of particular interest because it participates in ncRNA-directed TGS of the *PTEN* gene (67) and interacts with EZH2 at PRC2/3-repressed promoters (68), as well as with SETDB1 at several gene promoters, including the *RASSF1* promoter (69). DNMT3B also interacts with PRC2 and methylates the *RASSF1* promoter (70). The ChIP results showed that DNMT3A and DNMT3B localized to the *RASSF1* promoter, but not to the agRNA-targeted *AR* promoter (Figure 4C and D, respectively). The absence of DNMT3A and DNMT3B at the targeted *AR* promoter is consistent with the maintenance of a relatively low methylation state during TGS. Therefore, our results do not support the possibility that *de novo* DNA methylation ‘actively’ occurs at the agRNA-targeted *AR* promoter. In line with our results, it is worthwhile to note previous observations of the frequent association of H3K27me3 with DNA hypomethylation at promoters (71–73).

## DISCUSSION

Small RNA molecules bound to AGO proteins have been shown to modulate chromatin and affect gene expression by TGS. Early RNA-mediated TGS studies at the exogenous eukaryotic translation elongation factor 1 alpha (*EF1α*) promoter (20) and the endogenous E-cadherin (*CDH1*) promoter (21) implicated some epigenetic changes in promoter-targeted TGS. The former study showed that promoter-targeted agRNA-induced TGS was reversed by treatment with the histone deacetylase inhibitor trichostatin A or the DNA methyltransferase inhibitor 5-azacytidine (5-azaC). The latter showed that TGS was accompanied by enrichment of H3K9me2 in the promoter region. However, the mechanism(s) and related protein(s) responsible for the epigenetic changes have not been fully surveyed. Few proteins, including EZH2 (28,57,74), DNMT3A (22,67) and HDAC1 (29,74), have been shown to place themselves at agRNA-targeted promoters, as determined by ChIP analyses.

We have demonstrated that SETDB1 is involved in AGO2-mediated TGS of *AR* gene. The function of SETDB1 is essential, as knockdown of either SETDB1 or AGO2 abolished *AR* agRNA-induced TGS (Figure 1). Additionally, SETDB1-catalyzed H3K9me3 appears as well in the targeted *AR* promoter (Figure 2), and H3K27me3 was also observed. The *AR* gene was shown to be repressed by promoter-targeted agRNAs in a couple of studies (19,52), where histone modifications at the promoter were not analyzed, though. There are conflicting observations regarding histone modifications in connection with TGS. Some studies have reported that promoter-targeted TGS occurred independently of histone modification; in TGS of the *c-myc* gene, no substantial changes in H3K9me2 and H3K27me3 levels were observed in the promoter (75), and in TGS of the *PR* gene, no changes in H3K4me2 and H3K9me2 levels in the promoter were seen (52). This discrepancy between studies suggests either gene-specific histone modification mechanisms for promoter blockage, or the presence of other unknown mechanisms for TGS. A promoter turn-on/off mechanism without epigenetic alterations including histone modifications has yet to be proposed (76), and examination of all types of histone modifications is impracticable. Nevertheless, certain types of modifications could be considered important references for determining transcriptional productiveness of the target promoter, such as H3K9me3, which is thought to be one of the most important repressive chromatin markers (10,56). Thus, it is puzzling that changes in H3K9me3 levels have not been examined at agRNA-targeted promoters.

The agRNA-targeted *AR* promoter was modified by dual repressive marks: H3K9me3 by SETDB1 and H3K27me3 by EZH2. In support of their collaborative effect on TGS, SETDB1 was shown to be associated with EZH2 by IP (Figure 2H). This is intriguing as the SETDB1-EZH2 association indicates the coupling of H3K9me3 with H3K27me3 in promoter-targeted TGS. In embryonic stem cells (ESCs), promoters of genes that encode lineage-specific developmental regulators are occupied by PRC2 and contain nucleosomes with H3K27me3 (77–79), and a subset of these genes are also occupied by Setdb1 (80). Additionally, a significant overlap between Setdb1- and Suz12-bound sites was observed in mouse ESCs (81); Suz12, together with Ezh2, is a key member of the PRC2 complex. Such coexistence of dual repressive marks is not unusual and not limited to the H3K9me3-H3K27me3 match. Epigenomic studies in human ESCs, fetal lung fibroblasts (72) and mouse ESCs (80) have demonstrated that various combinations of H3K9me3, H3K27me3 and 5-mC repressive marks co-occupy promoters of many developmental regulatory genes, and that H3K9me3 and H3K27me3 together was most frequently observed. This multilevel epigenetic repression strategy might be advantageous in reducing the likelihood of escaping gene repression and altering cell fate.

It is unknown whether such a dual-lock device is a general mechanism in agRNA-induced promoter silencing, and whether each mark has a distinct function in agRNA-induced TGS. In certain chromatin contexts, H3K27me3 marks may not be sufficient for silencing, as they are often in a ‘bivalent’ state together with H3K4me3 in ESCs where ‘poised’ promoters are frequently leaky (77,82). A report observed that narrowly H3K27me3-marked promoters did not maintain stable silencing of gene expression until the H3K27me3-marked region was expanded several folds during differentiation (72). It would be interesting to determine whether such a dual-lock strategy is employed *in vivo*. If so, this dual lock to *AR* repression may reflect the developmental significance of AR, where leaky expression perturbs normal development and accounts for a wide range of pathological conditions (reviewed in (83)).

In *Arabidopsis thaliana*, agRNA-mediated TGS is associated with establishment of DNA methylation (84) catalyzed by DRM1, DRM2 and chromomethyltransferase 3 (85,86). In mammalian cells, however, the involvement of *de novo* DNA methylation in agRNA-triggered TGS is unclear. For example, agRNA targeting the *EF1α* promoter was shown to induce DNA methylation (20), whereas *CDH1* agRNA did not trigger DNA methylation of the promoter (21). A recent study showed that DNMT3A participated in ncRNA-directed TGS of the *PTEN* gene (67). These contradictory observations are likely due to the different cell models and experimental environments and/or different configuration of local chromatin (for example, different distribution and density of CpG dinucleotides) in gene promoters (87). At the silenced *AR* promoter, DNA methylation was not shown to occur extensively (Figure 4B). The low-level DNA methylation at the targeted *AR* promoter was similar to a previous study (19), and indicates that DNA methylation is not an on-off switch rapidly regulating transcriptional changes, and that transcriptional repression often precedes DNA methylation (88). If *de novo* DNA methylation activity had worked together with the AGO2-SETDB1 RITS complex, a dramatic increase in DNA methylation should have been observed. In support, ChIP results showed that DNMT3A and DNMT3B were not present at the targeted *AR* promoter (Figure 4C and D). Therefore, the results indicate that *de novo* DNA methylation activity does not participate in TGS of the *AR* promoter at least initially. The DNA methylation system may sense the silenced *AR* promoter ‘later’, as the epigenetic surveillance system recognizes the inertness of the targeted promoter and then recruits *de novo* methyltransferase(s) to more tightly guard the region against leaky expression.

**Figure 5.**
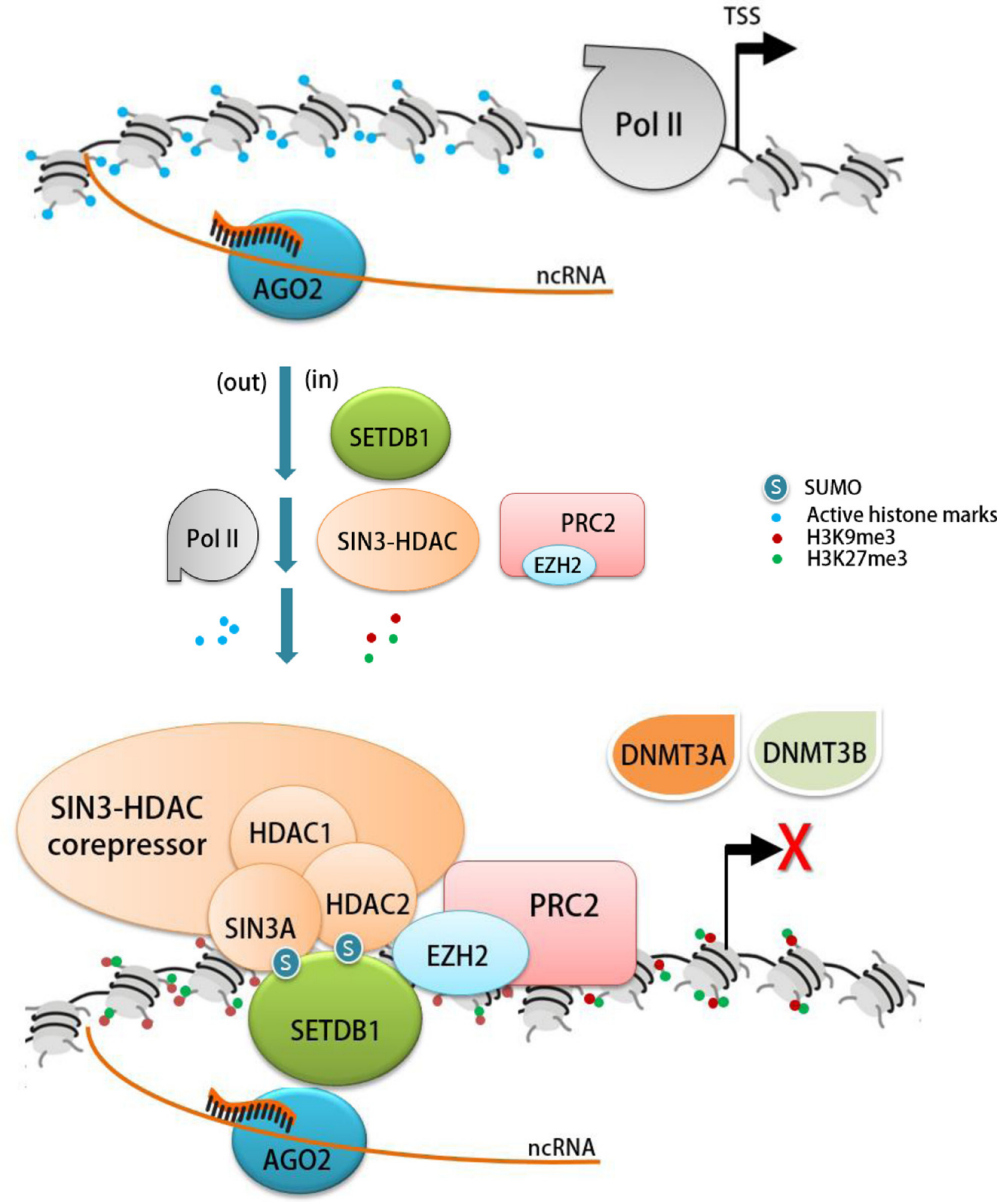
Model for AGO2-SETDB1-mediated silencing of the AR gene. agRNA-bound AGO2 traffics into the nucleus and binds to the target region of non-coding RNA. AGO2 then calls SETDB1 to position at neighboring chromatin (where SETDB1 may be either alone or form a platform) onto which the SIN3-HDAC remodeler can be tethered possibly through SUMO conjugation. The SIN3-HDAC complex creates a chromatin milieu unfavorable for transcription, i.e. through histone deacetylation. The ensuing establishment of H3K9me3 marks by SETDB1 and the addition of H3K27me3 by PRC2-EZH2 joining the multimeric protein complex ultimately evict RNA Pol II from the promoter, shifting the locus to transcriptionally inactive. This protein complex contains no *de novo* DNA methyltransferase activity (DNMT3A and DNMT3B).

We propose a molecular model to explain how the initial RITS complex of AGO2-SETDB1 silences the targeted *AR* promoter (Figure 5). AGO2 carrying agRNA enters the nucleus and moves to target ncRNA complementary to the agRNA expressed in the promoter. AGO2 then recruits SETDB1, which then binds neighboring chromatin using its double Tudor domains that can bind certain histone modifications including active histone marks (89). SETDB1 frequently uses SUMO to bind to diverse protein partners, such as Oct4 (38) and PML (51). SETDB1 itself possesses a number of potential sumoylation sites and SUMO interaction motifs that recognize conjugated SUMO (90), raising the possibility of a dimeric or multimeric SETDB1 assembly (not illustrated in the figure). SETDB1 then recruits the SIN3-HDAC chromatin remodeling complex and tethers it, possibly via SUMO interaction with SIN3A and HDAC1/2, as determined by their sensitivity to NEM (Figure 3A). The SIN3-HDAC corepressor complex removes acetylation and other active histone marks from surrounding chromatin, reversing the chromatin milieu and rendering it unfavorable for transcription. SETDB1 generates H3K9me3 marks at the promoter which, in general, serves to deposit HP1. It should be noted, however, that HP1 participating in TGS has not been demonstrated experimentally and remains to be explored. PRC2-EZH2 also joins the multimeric RITS complex and adds H3K27me3 to the *AR* promoter. *De novo* DNA methylating activity, such as DNMT3A and DNMT3B, is absent in the RITS complex (Figure 4C and D). Such alterations in histone modifications ultimately remove RNA Pol II from the promoter, marking the *AR* promoter unproductive for transcription.

Our results indicate that AGO2 interacts with SETDB1 and induces TGS through creating silent chromatin milieu at the targeted promoter. Studies of TGS in mammalian cells are still at an early stage compared with yeast and plant studies, and we expect our findings to expand knowledge of the mechanistic aspects of TGS in mammalian cells.

## SUPPLEMENTARY DATA

Supplementary Data are available at NAR Online.

SUPPLEMENTARY DATA
