# Supplementary material for: AGO2 and SETDB1 cooperate in promoter-targeted transcriptional silencing of the androgen receptor gene
Source: Nucleic Acids Res. 2014 Sep 2;42(22):13545–56. doi: 10.1093/nar/gku788 (PMC4267665; doi:10.1093/nar/gku788)
Supplement: SUPPLEMENTARY DATA [file supp_42_22_13545__index.html]

AGO2 and SETDB1 cooperate in promoter-targeted transcriptional silencing of the androgen receptor gene — AGO2 and SETDB1 cooperate in promoter-targeted transcriptional silencing of the androgen receptor gene — SUPPLEMENTARY DATA 

# AGO2 and SETDB1 cooperate in promoter-targeted transcriptional silencing of the androgen receptor gene

## SUPPLEMENTARY DATA

**Files in this Data Supplement:**

- SUPPLEMENTARY DATA
