# Supplementary material for: AGO2 and SETDB1 cooperate in promoter-targeted transcriptional silencing of the androgen receptor gene
Source: Nucleic Acids Res. 2014 Sep 2;42(22):13545–56. doi: 10.1093/nar/gku788 (PMC4267665; doi:10.1093/nar/gku788)
Supplement: SUPPLEMENTARY DATA [file supp_gku788_nar-01277-y-2014-File004.docx]

**Supplementary Table 1 – Information of the primers**

| **Analysis** | | **Primer** | **Position** | **5’-Sequence-3’** |
| --- | --- | --- | --- | --- |
| **Duplex RNA** | | M | - | UCUCUGGGCAGUCCAGAGCTT |
|  |  | AR | (–50)∼(–31) | UGGGGGCGGGACCCGACUCTT |
|  |  | PR | (–26)∼(–7) | AGGCGUUGUUAGAAAGCUGTT |
| **ChIP** | | AR-PP | (–189)∼(–21) | TTGTTTCTCCAAAGCCACTAGG  AACAGTTTGCGAGTCGGGT |
|  |  |  | (–660)∼(–450) | GAGTCTGGATGAGAAATGCATGGT  CTCCTACAAACTGAGGAGCAA |
|  |  | AR-DP | (–4170)∼(–3990) | GTAGAGGGAACAGAATATGCAA  TCCCCATTGCTCTTAATAGAAAA |
|  |  |  | (–3000)∼(–2840) | GAGCTAGTGATATACATAACAGCTTAAA  CCAATGATCTGCTTTCTGTCAGTATAGAC |
|  |  | PR-PP | (–419)∼(–290) | TGTTGGTCGCAGCAGGAGAA  GAGGAAGACATTTGGCTACATTATCTT |
|  |  | PR-DP | (–3000)∼(–2860) | AAAATCGTTTCCCAAAAGTAAAATC  CACTAATCTGCTTCTGTCACCAAATA |
|  |  | RASSF1 | (+11)∼(+150) | AGCCTGAGCTCATTGAGCTG  GCTCAGGCTCCCCCGACATG |
|  |  | Alu | - | TGGCGCGATCTCAGCTCACT  AGAGATGGAGACCATCCTGGCC |
| **RT-PCR** | Gene | AR | (+2000)∼(+2150) | AGACGACAGCGCAGGCAAGAG CAC  CAGGGTAGACGGCAGTTCAAGTGTCCC |
|  |  | PR | (+2500)∼(+2890) | GCTGTAAGGTCTTCTTTAAGAGGGC  ATTAAGACTTGTCAGCAAAGAACTGG |
|  |  | GAPDH | (+357)∼(+499) | GGCTGAGAACGGGAAGCTTGTCAT  CAGCCTTCTCCATGGTGGTGAAGA |
|  | ncRNA | F1 | (–501)∼(–480) | TATGACGGAATCTAAGGTTT |
|  |  | F2 | (–471)∼(–450) | TGCTCCTCAGTTTGTAGGAG |
|  |  | F3 | (–421)∼(–400) | ATCAGTCCTGAAAAGAACCC |
|  |  | F4 | (–281)∼(–260) | GGAGCCCTGGCGCCTAAACC |
|  |  | R1 | (–481)∼(–500) | AAACCTTAGATTCCGTCATA |
|  |  | R2 | (–451)∼(–470) | CTCCTACAAACTGAGGAGCA |
|  |  | R3 | (–401)∼(–420) | GGGTTCTTTTCAGGACTGAT |
|  |  | R4 | (–261)∼(–280) | GGTTTAGGCGCCAGGGCTCC |
|  |  | a1 | (–231)∼(–218) | AGATTGGGCTTTGGAACCAAATT |
|  |  | a2 | (–250)∼(–229) | TCCTGAATAGCTCCTGCTTTCC |
|  |  | b1 | (–181)∼(–160) | TGGGAGGTGGAGAGCAAATG |
|  |  | b2 | (–101)∼(–80) | TCCCACCTCCTTTTCCCTCC |
|  |  | b3 | (–31)∼(–10) | TGCGCCAGCACTTGTTTCTC |
| **DNA Methylation** | Bisulfite sequencing | M1 1^st^  PCR | (-459)∼(-308) | GTTTTAGTAAGTATTTGTTGGT  TTCCTAAAAACCAACACTCA |
|  |  | M1 2^nd^  PCR | (-431)∼(-335) | ATGGTTTGTTTTTTAGTTTGTA  TAATTCCAAAACCCAATCTA |
|  |  | M2 1^st^  PCR | (+496)∼(+704) | CGAGTTAGTTGTATATTGTA  ACTACTAAAAACCTAACTAC |
|  |  | M2 2^nd^  PCR | (+477)∼(+679) | TAAAGAAGGTTTTTAGGAGT  CATCTTTTAATCTCTAACTC |
